# Otogenic meningitis in children

**DOI:** 10.1007/s15010-025-02690-x

**Published:** 2025-11-17

**Authors:** Laura Lempinen, Riste Saat, Anu Laulajainen-Hongisto, Antti A. Aarnisalo, Tea Nieminen, Jussi Jero

**Affiliations:** 1 Department of Radiology, HUS Medical Imaging Center, Radiology, University of Helsinki and Helsinki University Hospital, PB 340, Helsinki, 00029 HUS Finland; 2 Head and Neck Surgery, Department of Otorhinolaryngology, University of Helsinki and Helsinki University Hospital, Helsinki, Finland; 3 Radiology, East Tallinn Central Hospital, Tallinn, Estonia; 4 Children’s Hospital, Department of Paediatrics, University of Helsinki and Helsinki University Hospital, Helsinki, Finland; 5 University of Helsinki and Helsinki University Hospital, Helsinki, Finland

**Keywords:** Otogenic meningitis, Otitis media, Post-meningitis sequela, Hearing loss, Deafness, Children

## Abstract

**Purpose:**

Otogenic meningitis is a rare but potentially life-threatening intracranial complication of otitis media (OM). Our aim was to study the incidence of childhood otogenic meningitis and to compare clinical presentation, causative pathogens, diagnostics, treatment, and outcome of otogenic versus non-otogenic meningitis.

**Methods:**

Charts were reviewed for 47 children admitted to our tertiary center with bacterial meningitis (BM) between 2010 and 2020. Otoscopy and/or imaging were used to determine the otogenic meningitis ratio and the mean annual incidence was calculated.

**Results:**

Eight (17%) of the 47 BM cases were otogenic [5 males; median age 1.3 years (range 2 months to 16 years)]. The otogenic meningitis incidence was 0.3/100 000/year. The classic triad of fever, altered level of consciousness, and meningeal irritation was more common in children with otogenic meningitis (50%, 4/8) than without OM (14%, 5/36) (*P = 0.042*). *Streptococcus pneumoniae* was a more common pathogen in children with OM (88%, 7/8) than without OM (14%, 4/29) (*P < 0.001*), whereas *Neisseria meningitidis* infection occurred only in children without OM (41%, 12/29) (*P = 0.036*). Neurological sequelae at discharge were present in 3 (38%) children with OM. Deafness was diagnosed in two children, both with otogenic backgrounds. Three children showed long-term sequelae: 2 had deafness (aged < 2 years) and 1 had aphasia/dysphasia.

**Conclusion:**

The incidence of otogenic meningitis was 0.3/100 000/year, with *S. pneumoniae* the most common causative pathogen. Deafness was the most common long-term sequela and occurred only in children with otogenic meningitis.

## Introduction

Otitis media (OM) is a common childhood infection with a mostly benign clinical course [1]. However, in rare cases, OM infection can spread intracranially. Otogenic meningitis is the most common intracranial complication (30–72%) of OM [2–4], and up to 45% of bacterial meningitis (BM) cases in children may occur secondary to OM [4, 5]. Nevertheless, only few studies have analyzed the prevalence of concomitant ear infection in cases of bacterial meningitis (BM) [2, 4, 6]. Otogenic meningitis is potentially life-threatening and can cause significant morbidity [4, 7, 8]; thus, early diagnosis is important for initiating appropriate treatment.

BM remains a severe disease, especially in children, where it has a 3–9% fatality rate. Furthermore, 18–49% of the survivors of BM have a high risk of neurological sequelae, such as hearing loss, cognitive impairment, motor weakness or paralysis, incoordination, and seizures [9–11], with hearing loss the most common BM sequela [4, 9, 12]. The most common BM-causing pathogens are *Streptococcus pneumoniae* and *Neisseria meningitidis* [10]. *S. pneumoniae* is also the most common pathogen causing OM [13], and pneumococcal meningitis is associated with a greater risk for hearing loss and severe outcomes [10, 12, 14]. Patients with otogenic meningitis also tend to have less favorable outcome [6, 14] and are at greater risk for long-term hearing loss [12].

Our aim in the present study was to investigate the incidence of otogenic meningitis in children over an 11-year period and to compare its clinical presentation, causative pathogens, and outcome to those with non-otogenic meningitis.

## Materials and methods

### Study design and study population

Our study analyzed retrospectively the medical records of all children aged 2 months to ≤ 16 years, who were treated for BM at our tertiary referral center at Helsinki University Hospital, Department of Children and Adolescents, from January 2010 to December 2020. The catchment population (≤ 16 years of age) in the Hospital District of Helsinki and Uusimaa increased from 278 320 in 2010 to 295 525 in 2020 during this time. The mean catchment population over the 11-year period was 288,637.

**Fig. 1:**
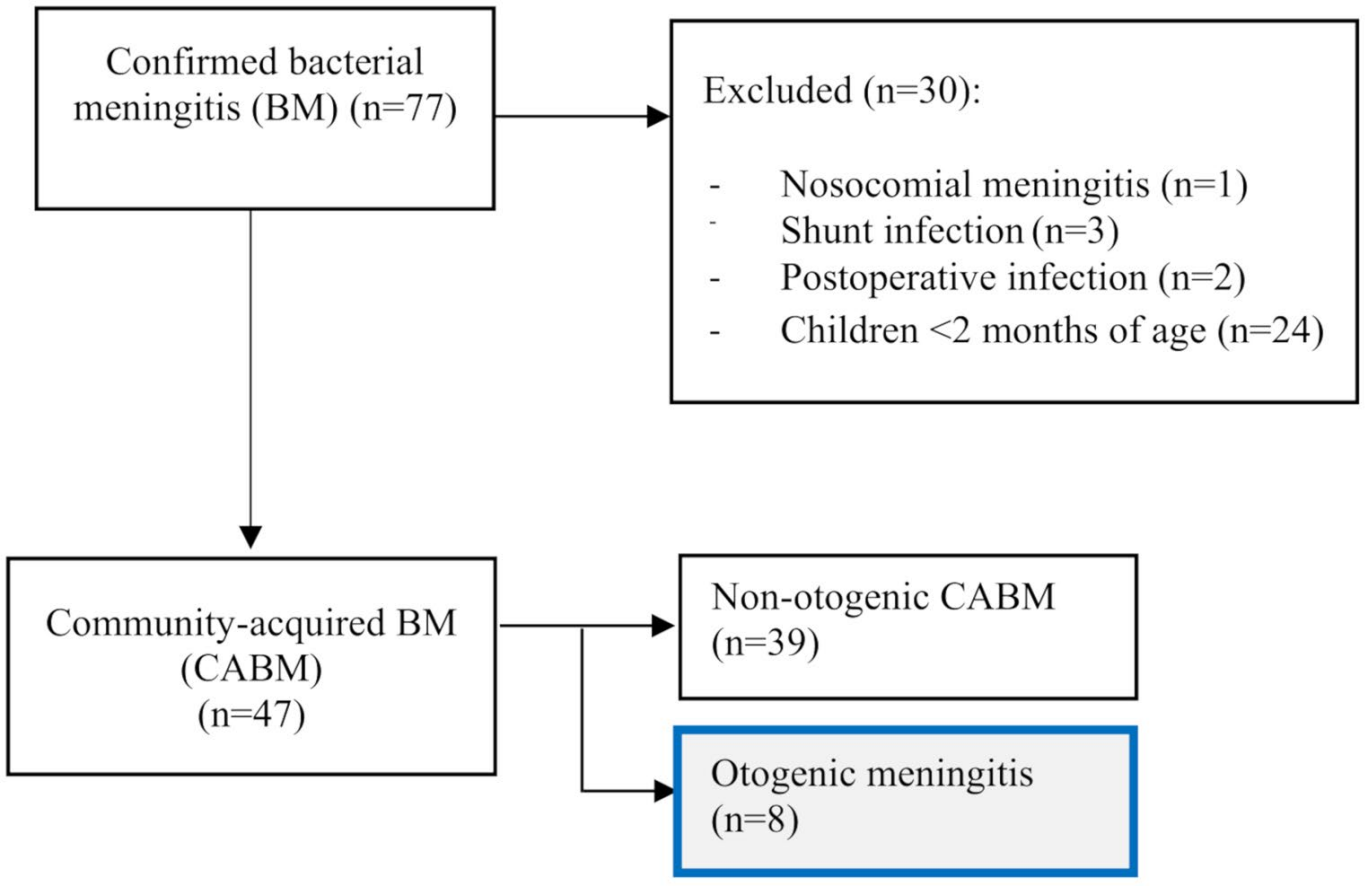
Study flow chart

Patient data were obtained from the hospital’s electronic database. We used the international Classification of Diseases version 10 for our patient data search and codes G00.0–G00.3, G00.8, G00.9, G01*, G01*A01.0, G01*A02.2, G01*A17.0, G01*A22.8, G01*A32.1, G01*A39.0, G03, G03.0-G03.2, G03.8, G03.9, A39.0, G05.0*A17.8, G05.0*A32.1, and G05.0*A39.8. The methods and analysis of the entire study sample (*n* = 77 BM cases) have been published previously [15]. Community-acquired BM was defined as BM acquired outside of the hospital. No patient had a history of neurosurgery within the previous 30 days. Nosocomial BMs, neuroborreliosis, or meningitis caused by non-bacterial pathogens (parasite, virus, or fungi) were excluded. All BM cases in our data were community acquired (Fig. 1). This study focused on the 8 cases (17%) from this BM cohort (*n* = 47) who showed predisposing middle ear infection and compared the clinical presentation, causative pathogens, and outcomes to cases of non-otogenic childhood BM.

### OM diagnosis

In our cohort, 34 of the 47 children (72%) had a record of otoscopy. The OM was diagnosed by otoscopy (*n* = 3), by magnetic resonance imaging (MRI) (*n* = 3), and by otoscopy and MRI (*n* = 2). According to the imaging findings, acute mastoiditis (AM) was defined as total or near-total mastoid obliteration, together with intramastoid enhancement or diffusion restriction. OM was defined as at least 50% mastoid fluid retention and mild intramastoid enhancement in MRI. Labyrinthitis was defined as labyrinth enhancement on T1-weighted MRI with gadolinium contrast [16, 17]. In total, we lacked the middle ear status in 5 (11%) children diagnosed with BM.

All computed tomography (CT) and MRI studies of the head and neck region were reassessed by two radiologists (RS and LL) for neuroradiological findings and otogenic infections. The radiologists’ reports of brain ultrasound examinations were also inspected for intracranial complications. The following conditions were defined as neuroradiological complications: hemorrhage; arterial or venous infarction; newly acquired hydrocephalus; pus in the subdural space, subarachnoid space, or lateral ventricles; subdural effusion/collection; labyrinthitis; brain herniation; cerebral edema; spinal epidural abscess; and encephalitis.

Outcome was assessed according to the Glasgow Outcome scale (GOS) at discharge as the following: fatal outcome (1 point); vegetative state (2 points); severe disability, dependent on others for activities of daily living (3 points); moderate disability, independent in daily living but with neurological deficit making the person unable to return to school (4 points); and mild or no disability and resumption of normal daily life (5 points) [18]. Unfavorable outcome was classified as a GOS score of 1–4.

Long-term neurological sequelae were defined as persistent symptoms after discharge, diagnosed either during the hospital stay or follow-up. This included dysphasia/aphasia, hearing loss, deafness with cochlear implantation, dizziness, and learning difficulties. The median follow-up time was 11 months (range 1 month to 9 years). After specialized healthcare monitoring, follow-up continued at child welfare clinics for preschoolers and school health services for school-aged children.

Hearing was tested using otoacoustic emissions (*n* = 21), audiometry (*n* = 6), behavioral observation audiometry (BOA) (*n* = 13), and auditory brainstem response (ABR) (*n* = 1). Hearing loss was deemed if the hearing threshold was > 25 decibels Hearing Level (dB HL) and was further subdivided into moderate (41–60 dB HL), severe (61–80 dB HL), and profound/deaf (> 80 dB HL). The hearing thresholds are presented according to the better-ear hearing level (BEHL) or ear-specific.

The permission for this retrospective study was obtained from the Helsinki University Central Hospital Pediatric Research Center. Approval from the local Ethics Committee was not required for this type of study at the time the study was conducted.

### Statistical analysis

Statistical analysis was performed using SPSS Statistics version 29.0 software (Armonk, NY: IBM Corp). Fisher’s exact test or the χ2 test was used when appropriate to determine the significance of the categorical variables. The Mann-Whitney *U* test was used to analyze the correspondence of medians with the interquartile ranges (IQR) of continuous variables. Two-tailed *P* values of *< 0.05* were regarded as statistically significant.

**Table 1:** Clinical findings, symptoms and etiology in children diagnosed with otogenic meningitis, 2010–2020

Case	Year	Age / gender	Type of ear infection	Duration of symptoms (days)	Symptoms and signs	Pathogen, serotype	Intensive care
1	2011	1,6 y /M	OM (L)	3	Fever, nausea/vomiting, coma, seizures, any psychomotor retardation.	*Pnc*, 23 F	No
2	2013	1,1 y /M	AM (R, L)	3	Fever 39,4 °C, diarrhea. altered level of consciousness, any psychomotor retardation, meningeal irritation.	Pnc*,10 A	Yes
3	2013	1,6 y /F	OM (L)	3	Flu symptoms, fever 39,0 °C, nausea/vomiting, disorientation, altered level of consciousness, any psychomotor retardation, meningeal irritation, motor delay/hypotonia.	Pnc*, 19 F	Yes
4	2014	5,2 y /M	OM (R)	1	Headache, nausea/vomiting, fever 39,7 °C, meningeal irritation, Petechia.	*Pnc*, 14	No
5^a^	2017	1,6 y /F	OM and AM (R)	3	Fever 38,6 °C, nausea/vomiting, altered level of consciousness, meningeal irritation.	Pnc*, 6 C	Yes
6	2018	0,4 y/M	OM and AM (R)	5	Fever 40 °C, nausea, bad appetite, irritable.	Pnc *, 35 F	Yes
7	2018	0.2 y /M	OM (L)	1	Irritable, fever, skin has marbled appearance.	G + cocci	No
8	2020	0,8 y /F	OM (L)	3	Nausea/vomiting, fever 39,8 °C, irritable, petechia, seizures, declined general condition.	*Pnc*, 10 A	No

Abbreviations: F, Female; Gram positive cocci; G + cocci; L, left; Male, M; *Pnc*,* Streptococcus pneumonia*; R, right; y, year

*Had had pneumococcal vaccination (covering *Streptococcus pneumoniae* serotypes 1, 4, 5, 6B, 7 F, 9 V, 14, 18 C, 19 F &23 F)

^a^ Patient had bacterial meningitis in 4/2016 and 3/2017

## Results

### Age, demographics, and medical history

Eight children with BM were diagnosed with OM/AM (Tables 1 and 2). Five of these (63%) were male, and the median age was 1.3 years (range, 2 months to 5.2 years). Five of the 8 patients with otogenic meningitis also had mastoiditis. No cholesteatoma or chronic otitis media was present. The incidence of otogenic meningitis was 0.3/100 000/year.

**Table 2:** Neurological symptoms, imaging findings and outcome of otogenic meningitis

Case	Neurological symptoms during hospital stay	Surgical treatment	Imaging findings	Neurological sequelae at discharge	Long-term neurological sequalae and final hearing
1	Mild strabismus (R), profound hearing loss/deaf, dizziness.	Tympanostomy (L). Bilateral cochlear implantation	Bilateral labyrinthitis	Deaf and dizziness	Deaf/cochlear implantation
2	Motor delay/isolated hypotonia	Bilateral cochlear implantation	Bilateral cochlear fibrosis	Deaf, dizziness, and motor delay/isolated hypotonia	Deaf/cochlear implantation
3	Coma (intubated), mild oculomotorius nerve paresis	No	No imaging	No	No
4	Dizziness, altered level of consciousness	No	Leptomeningeal enhancement	No	No
5	No neurological symptoms.	Tympanostomy (bilateral)	Brain ischemia, pachymenigeal, mild cranial nerve enhancement.	No	No
6	Seizures, abducens nerve paresis (L), altered level of consciousness	Tympanostomy (bilateral)	Meningeal thickening (Brain US)	Left hemiparesis and convulsions	Unilateral mild hearing loss
7*	No neurological symptoms.	Tympanostomy	Normal (Brain US)	No	No
8	Coma (intubated)	Revision of avascular necrosis of distal extremities	No imaging.	No	Delayed speech development. Hearing not tested.

Abbreviations: L, left; R, right; US, ultrasound

Among the children with otogenic meningitis, the median duration of symptoms before hospitalization was 3 days (IQR 1.5–3 days). Five of the 8 children with otogenic meningitis had nausea/vomiting before hospital admission. None complained of ear symptoms. At admission, meningeal irritation and fever > 37.9 °C were common, occurring in 5/6 (83%) children with OM/AM. Disorientation occurred in 3/8 (38%), petechiae in 2/8 (25%), and seizures and cranial nerve palsy in 1/8 (13%) of the children with otogenic meningitis. The classical triad of fever, altered level of consciousness, and meningeal irritation at hospitalization was more commonly diagnosed in children with OM/AM (50%, 4/8) than in children with without OM/AM (14%, 5/36) (*P = 0.002*). (Table 3)

Table 4 summarizes the etiology of BM determined from cerebrospinal fluid (CSF) samples. The most common pathogen in otogenic meningitis was *S. pneumoniae* (*n* = 7, 88%) (*P < 0.001*), followed by gram-positive cocci (*n* = 1). The serotypes of *S*. *pneumoniae* were 10 A in 2 of the 8 patients, and 6 C, 14, 19 F, 23 F, and 35 F in 1 patient each. *N. meningitidis* was not found in children with otogenic meningitis, whereas it was the most common pathogen causing non-otogenic meningitis (31%, 12/39) (*P = 0.036*).

**Table 3:** The baseline characteristics of children diagnosed with bacterial meningitis with or without otitis media (OM) /acute mastoiditis (AM)

	All *N* = 47 (%) or median (IQR)	without OM/AM *N* = 39 (%) or median (IQR	with OM/AM *N* = 8 (%) or median (IQR)
Males	29 (62)	24 (62)	5 (63)
Age (years)	1.6 (0.4–9.8)	1.6 (0.4–11.6)	1.3 (0.5–1.7)
Previous antibiotic	11 (23)	9 (23)	2 (25)
Upper respiratory tract infection symptoms	22 (47)	20 (51)	2 (25)
Nausea/vomiting	30 (64)	25 (64)	5 (63)
**Clinical and laboratory findings on admission**			
Fever > 37,9 °C (*n* = 34)	22 (47)	17/28 (61)	5/6 (83)
Headache (*n* = 24)	10 (21)	10/20 (50)	0/8
Petechia (*n* = 43)	11 (23)	9/35 (26)	2/8 (25)
Meningeal irritation (*n* = 44)	16 (34)	11/36 (31)	5/8 (63)
Triad (fever, altered consciousness and meningeal irritation) (*n* = 44)	9 (19)	5/36 (14)	4/8 (50).^*0.042*^
Any neurological sequelae ^b^ (*n* = 45)	16 (34)	14/37 (38)	3/8 (38)
Seizure (*n* = 45)	4 (9)	3/37 (8)	1 (13)
Altered level of consciousness (*n* = 45)	14 (30)	11/37 (30)	3 (38)
Coma (*n* = 45)	4 (9)	3/37 (8)	1 (13)
C-reactive protein (mg/l), Day1 (*n* = 45)	97 (53–199)	88 (53–201)	166 (79–200)
B-Leukocytes (x10^9^), Day1 (*n* = 45)	15.1 (8.1–22.3)	14.6 (7.4–20.8)	21.6 (9.8–24.8)
CSF leucocyte count (x10^6^/L), Day1 (*n* = 43)	1130 (120–3200)	1340 (117–2630)	655 (125–5320)
CSF protein (g/L), Day1 (*n* = 39)	1291 (702–3140	1204 (629–1204)	2042 (1116–3179)
CSF glucose (mmol/L), Day1 (*n* = 37)	2.9 (1.0-4.1)	3.1 (1.6–4.1)	1.6 (0.2–4.1)
CSF culture detection of pathogen	35 (74)	27 (69)	8 (100)
Blood culture positive	27/43 (63)	23/37 (62)	4/6 (67)
Number of patients with neuroradiological complication (*n* = 39)	19/39 (49)	16/32 (50)	3/7 (43)
Operation done, number of patients	10 (21)	5 (13)	5 (63) ^*0.007*^
Tympanostomy	3 (6)	0	*3 (38)* ^*0.003*^
Cochlear implantation (bilateral)	2 (4)	0	2 (25) ^*0.026*^
Intensive care unit admission	20 (43)	16 (41)	4 (50)
Adjunctive anticonvulsant treatment	6 (13)	5 (13)	1 (13)
Adjunctive corticosteroid treatment	23 (49)	21 (54)	2 (25)

Statistical significance when *P* < 0.05

**Table 4:** Bacterial etiologies of children diagnosed with bacterial meningitis with or without otitis media (OM) /acute mastoiditis (AM)

Pathogen	All *N* = 47 (%)	without OM/AM *N* = 39 (%)	with OM/AM *N* = 8 (%)
No growth	12 (26)	12 (31)	0/8
*Neisseria meningitidis*	12 (26)	12 (31)	0/8 ^*0.036*^
*Streptococcus pneumonia*	11 (23)	4 (10)	7 (88) ^*<0.001*^
*Haemophilus influenzae type* b	2 (4)	2 (5)	0/8
Group B *streptococcus* (GBS)	2 (4)	2 (5)	0/8
*Staphylococcus aureus* ^b^	3 (6)	3 (8)	0/8
*Escherichia coli*	2 (4)	2 (5)	0/8
Gram positive cocci	1 (2)	0/39	1 (13)
*Mycobacterium tuberculosis*	2 (4)	2 (5)	0/8

Statistical significance when *P* < 0.05

### Treatment of otogenic BM

The median duration of hospital stay was 9 days (IQR 7–12). Half (*n* = 4, 50%) of the children with otogenic meningitis were admitted to the intensive care unit during their illness. Adjunctive anticonvulsant treatment was administered to one child and corticosteroid treatment to two children with otogenic meningitis.

A total of 6 operations were performed after discharge on 5 of the 8 children with OM/AM within a median of 2 months (range 1 to 3 months) after their BM diagnosis. Surgery was more frequent in the children with otogenic meningitis (63%, 5/8) than in the children without OM/AM (13%, 5/39) (*P* = 0.007). Bilateral cochlear implantation was more common in patients with otogenic meningitis (25%, 2/8) versus non-otogenic meningitis (0%) (*P* = 0.026). A bacterial culture was obtained in only one tympanostomy and yielded a negative result. No mastoidectomies were performed.

### Radiological findings

**Fig. 2:**
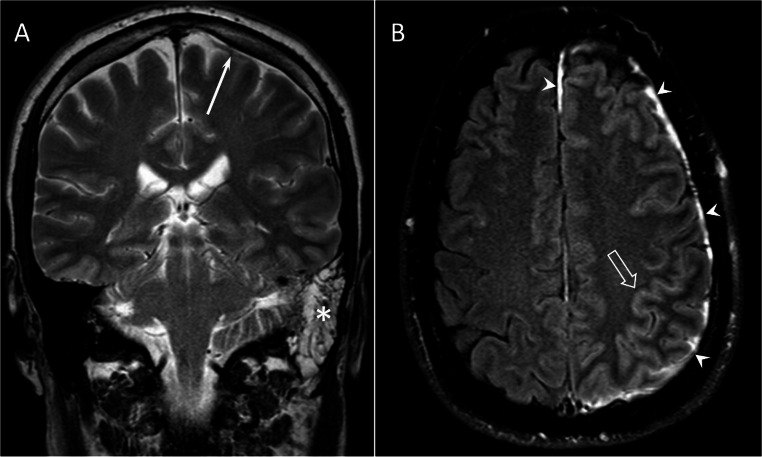
Otogenic meningitis on MRI. Obliteration of the left mastoid (asterisk) and meningeal thickening (arrow) on coronal T2 (**A**). Enhancement of the dura (arrowheads) and cerebral sulci (open arrow) on axial Gd FLAIR (**B**)

Overall, 39/47 (83%) of the children with BM underwent medical imaging, including CT (*n* = 13), MRI (*n* = 27), and brain ultrasound (*n* = 17). Conversely, 7/8 (88%) of the children with otogenic meningitis underwent medical imaging. MRI of the ear was performed on two patients (both became deaf and had bilateral cochlear implants). CT of the ear was performed in one child with right-sided mastoiditis due to recurrent BM infection. Neuroradiological complications were diagnosed in 3/8 (38%) of the scanned patients with otogenic meningitis; these were labyrinthitis (*n* = 2) and brain infarction (*n* = 1). Figure 2 shows MRI findings in otogenic meningitis.

### Hearing results in children with otogenic vs. non-otogenic meningitis

Hearing was tested primarily after hospitalization in 30 of the 47 children; only four children received a hearing test and ENT consultation during their hospital stays. The first hearing test after BM diagnosis was performed earlier in the otogenic vs. the non-otogenic group (a median of 3 weeks vs. 2 months) (*P = 0.032*).

Hearing loss was diagnosed in 4 (13%) children with BM according to hearing thresholds by ear; all 4 had labyrinthitis findings on MRI. Bilateral deafness was diagnosed in 2 with dizziness; both had otogenic backgrounds (25%, 2/8) (*P = 0.044*) and pneumococcal etiology (serotypes were 23 F and 10 A). One case of unilateral deafness was caused by *N. meningitidis* in a child without OM. Another child without OM experienced dizziness and had a hearing threshold of 32/45 dB. However, during long-term follow-up, the hearing threshold normalized, and the dizziness resolved. In our cohort, possible fluctuation in hearing levels was present in 5/30 (17%) of the children with BM.

### Clinical outcomes

At discharge, neurological sequelae of any type occurred in 3/8 (38%) of the children with otogenic meningitis; these sequelae included deafness (*n* = 2), dizziness (*n* = 2), motor delay/isolated hypotonia (*n* = 2), seizures (*n* = 1), and hemiparesis (*n* = 1). No fatalities occurred among the children with otogenic background. At discharge, severe disability (GOS 3) was diagnosed more frequently in children with otogenic meningitis (38%, 3/8) than with non-otogenic BM (5%, 2/39) (*P = 0.029*).

In the follow-up, long-term sequelae were diagnosed in 3/8 (38%) children with otogenic meningitis. The long-term sequelae were deafness (25%, *n* = 2) and aphasia/dysphasia (13%, *n* = 1). The median of follow-up was 2.5 years (range 4 months to 9 years). (Table 5)

**Table 5:** Outcome of children diagnosed with bacterial meningitis with or without otitis media (OM) /acute mastoiditis (AM)

	All Children *N* = 47 *n*/*N* (%)	Children without OM/AM *N* = 39 *n*/*N* (%)	Children with OM/AM *N* = 8 *n*/*N* (%)
**Glasgow Outcome Score (GOS) at discharge**			
GOS 1	5 (11)	5 (13)	0/8
GOS 2	0/47	0/39	0/8
GOS 3	5 (11)	2 (5)	3 (38) ^*0.029*^
GOS 4	1 (2)	1 (3)	1 (13)
GOS 5	36 (77)	31 (79)	5 (63)
Unfavorable outcome (GOS 1–4)	11 (23)	8 (21)	3 (38)
Any neurological sequelae	14/42 (33)	11/34 (32)	3 (38)
Dizziness (*n* = 34)	7/34 (21)	5/26 (19)	2 (25)
Motor delay/isolated hypotonia	5/42 (12)	3/34 (9)	2 (25)
Seizures	3/42 (7)	2/34 (6)	1 (13)
Hemiparesis	2/42 (5)	1/34 (3)	1 (13)
Deafness	2/42 (5)	0/34	2 (25)
Mild abducens nerve paresis	1/42 (2)	1/34 (3)	0/8
Any psychomotor retardation	1/42 (2)	1/34 (3)	0/8
Aphasia/dysphasia	2/42 (5)	2/34 (6)	0/8
**The long-term follow-up**	**N = 36**	**N = 28**	**N = 8**
The follow-up, median in years (range)	0.9 (0.02–9.3)	0.9 (0.02–8.3)	2.5 (0.3–9.3)
Long-term neurological sequelae	9 (25)	6 (21)	3 (38)
Aphasia/dysphasia	4 (11)	3 (11)	1 (13)
Learning difficulties	2 (6)	2 (7)	0/8
Dizziness	1 (3)	1 (4)	0/8
Normal hearing (BEHL $\:\le\:$26 dB HL)	28/30 (93)	28 (100)	6/8 (75)^*0.044*^
Hearing impairment/deafness	2/30 (7)	0/20	2 (25) ^*0.044*^

Abbreviations: BEHL, better ear hearing level; dB, decibel; HL hearing level

Statistical significance when *P* < 0.05

## Discussion

In this retrospective cohort study of children with BM over 11 year-period in Southern Finland, 17% of the BM cases had an otogenic origin. Despite national guidelines, otoscopic examinations were not performed for all BM patients [2, 6]. In our cohort, 34/47 (72%) children had records of otoscopy. In our cohort, one-third of the children’s ears were not examined despite national guidelines recommending such assessment. This issue has also been noted by Swedish study group, who reported that otoscopic examinations are not performed for all bacterial meningitis patients. The Bjar et al. found that only 54% (116 of 216) had any record of otoscopy or otomicroscopy [2]. In this light, the incidence of otogenic meningitis may be underrecognized in literature. For 3 children, the concomitant OM/AM was inferred from MRI findings.

Our data cohort is small, but it still provides some perspective regarding the incidence of childhood otogenic meningitis. Children with otogenic meningitis may not present with clear otologic signs, which may delay diagnosis and treatment. Notably, none of the children with otogenic meningitis in our study complained of ear pain, likely due to their young age (median age of 1.3 years). Nausea or vomiting were the most frequent symptoms, occurring in 5/8 (63%) of the children with otogenic meningitis before hospitalization, thereby emphasizing the importance of checking middle ear status in all sick children [2]. The available literature indicates that nausea and/or vomiting is a frequent (74–91%) symptom in children diagnosed with BM [9, 14], whereas vomiting is less common in OM, occurring in 4–9% in children and neonates [19, 20]. Our data indicates a greater prevalence of vomiting/nausea in our otogenic children than has been reported in previous studies.

The incidence of otogenic meningitis was 0.3/100 000/year in our cohort, which is in line with a study from the United Kingdom [21]. Previously published studies provided only scanty data regarding the proportion of concomitant OM in cases of childhood BM or about the clinical findings and outcomes of otogenic versus non-otogenic meningitis. Two Danish studies reported that 21% and 45% of children with BM (< 16 years of age) had concomitant OM [4, 5]. These results are quite in line with our finding that 17% of our BM cases had an otogenic origin.

In our study, *S. pneumoniae* was the leading pathogen detected in CSF cultures in 88% of patients with otogenic meningitis. Only one child with an otogenic background had other Gram-positive cocci in the CSF. Bjar et al., in a study that ran from 2000 to 2017, reported that *S. pneumoniae* was the most common pathogen identified in CSF in all age groups of children < 16 years of age, whereas *N. meningitidis* was predominant in teenagers [2]. According to the Finnish national vaccination program, the *Haemophilus influenzae* type b (Hib) vaccines have been administered at the ages of 3, 5, and 12 months since 1993, while the 10-valent pneumococcal conjugate vaccine (PCV10), which covers the *S. pneumoniae* serotypes 1, 4, 5, 6B, 7 F, 9 V, 14, 18 C, 19 F, and 23 F. PCV10 has been given following the same schedule as the Hib vaccine since the autumn of 2010. Interestingly, three children in our cohort with otogenic meningitis had *S. pneumoniae* serotypes 23 F, 19 F, and 14, which should have been covered by PCV10. Unfortunately, vaccination status was mentioned only in the part of medical records. Four children with otogenic meningitis had a note in their medical records indicating receipt of PCV before their current illness.

In our study, we noted that 4 of 7 (57%) otogenic pneumococcal serotypes were non-vaccine-type. PCV vaccines are effective against invasive pneumococcal disease, and they have caused a decline in tympanostomies in children [22, 23]. The pneumococcal serotypes causing deafness are 23 F and 10 A, but serotype 10 A is not included in PCV10. A recent pneumococcal meningitis study reported that 23 F serotype caused hearing loss in 10% of adult patients with BM [24]. In general, however, the prevalence of hospital admissions for pediatric acute otitis media/otogenic complications and pneumococcal meningitis associated with otitis media has declined since the introduction of pneumococcal vaccination [25].

Hearing loss was the most common sequela after BM in our cohort, as reported in the literature. However, in our data, the incidence of post-meningitis hearing loss in children was surprisingly low (10%) when compared with rates of 14–35% in previous reports [4, 9, 10, 26]. In the long-term follow-up of 36 children with BM, only 2 (7%) were diagnosed with deafness and both had otogenic meningitis. During follow-up, no cases of severe, moderate, or mild hearing loss were identified in our data. As previously published, bilateral severe to profound hearing loss has been reported in approximately 1–5% of BM survivors, which is in line with the 7% detected in our study [12, 27]. Hearing loss is usually associated with *S. pneumoniae* [9, 26–28], as was the case with both of our patients with profound hearing loss. Hearing rehabilitation plays a crucial role in every child’s social and educational development.

In our study, hearing was tested in 64% of the children, which is less than ideal. This needs to be given more attention in the future. Although national guidelines recommend hearing tests for children with BM, this is not always followed in practice. Monitoring hearing after diagnosis of BM is crucial because associated bacterial labyrinthitis may lead to partial or total ossification of the labyrinth, making subsequent cochlear implantation very challenging even as soon as 2 months of diagnosis [29]. This was the case in our two patients with profound hearing loss. If cochlear implantation seems likely, imaging should be performed promptly, as the best results using cochlear implants for a deaf ear are obtained when the procedure is performed without significant delay [30]. Performing hearing tests within 2 weeks of a BM diagnosis would be advisable.

In cases of otogenic meningitis, tympanostomy was chosen for 3 out of 8 patients (38%), with all procedures performed after discharge. The timing and necessity of surgical intervention have become contentious topics, with some sources recommending urgent mastoidectomy, while others suggesting myringotomy as the only required procedure [7, 31]. In our study, no mastoidectomies were performed. Earlier, in cases of intracranial complications of OM, mastoidectomy was the gold standard of care [31]. One animal study showed that hearing loss in pneumococcal meningitis is linked to the bacterial load in the middle ear, suggesting that draining middle ear infections may reduce the risk of hearing loss [32]. This underscores the importance of thorough ear examinations in children diagnosed with BM. Early ear, nose and throat (ENT) consultation is crucial, highlighting the importance of performing myringotomy or tympanostomy with culture at the onset of otogenic disease. Based on our experience with otogenic meningitis, we recommend ENT consultation and performing myringotomy/tympanostomy, as well as considering mastoidectomy in case of complications and strong disease symptoms.

Our study’s main limitation was its retrospective nature, but the treatment protocol for BM is considerably standardized in our tertiary center. Another limitation was the small sample size, which was influenced by the country’s small population and the low incidence of BM. Also, 5 of the 47 children with BM were lacking an ear status. Our data cohort is small, but it provides a perspective on the incidence of childhood otogenic meningitis. As a strength, our study adds to the presently scanty data that has been published previously regarding otogenic versus non-otogenic BM. European studies on otogenic meningitis fail to provide information on how the diagnosis of otitis media was established (whether by otoscopy / otomicroscopy / ENT consultation / CT / MRI), nor do they specify how many patients had their middle ear status evaluated [4, 5, 33]. Considering this, our results provide important context when assessing the reported incidence of otogenic meningitis in children.

## Conclusion

This cohort study, conducted over an 11-year period, revealed that 17% of the pediatric BM cases had an otogenic background. The incidence of otogenic meningitis was 0.3/100 000/year, and *S. pneumoniae* was the leading pathogen (88%). Of all children diagnosed with BM, otoscopic examination was lacking in almost one-third, and hearing was tested only in 64% of these patients. Early ENT consultation is crucial to manage the clinical course of otogenic meningitis. Only two children with otogenic meningitis were diagnosed with hearing loss; both had a pneumococcal etiology and became deaf.

## Data Availability

Study data can be provided upon reasonable request by the corresponding author.

## Abbreviations

ABR: Auditory brainstem response
AM: Acute mastoiditis
BOA: Behavioral observation audiometry
BM: Bacterial meningitis
BEHL: Better ear hearing level
CABM: Community acquired bacterial meningitis
CT: Computed tomography
CSF: Cerebrospinal fluid
*dB HL*: Decibels Hearing Level
ENT: Ear, nose and throat
GBS: Group B streptococcus
GSC: Glasgow Coma Scale
GOS: Glasgow Outcome Scale
IQR: Interquartile range
Hib: *Haemophilus influenzae* type b
HUS: Helsinki and Uusimaa Hospital District
LO: Labyrinthitis ossification
*N. meningitidis*: *Neisseria meningitidis*
OAE: Otoacoustic emission
OM: Otitis media
PCV: Pneumococcal conjugate vaccine
Pnc: Streptococcus pneumoniae
MRI: Magnetic resonance imaging
*S. pneumoniae*: *Streptococcus pneumoniae*
